# Irinotecan Plus Mitomycin C as Second-Line Chemotherapy for Advanced Gastric Cancer Resistant to Fluoropyrimidine and Cisplatin: A Retrospective Study

**DOI:** 10.1155/2012/640401

**Published:** 2012-02-15

**Authors:** Kohei Ogawa, Ayumu Hosokawa, Akira Ueda, Seiko Saito, Hiroshi Mihara, Takayuki Ando, Shinya Kajiura, Mitsuhiro Terada, Yuji Tsukioka, Naoki Horikawa, Takashi Kobayashi, Masayuki Note, Kunihiro Sawasaki, Junya Fukuoka, Toshiro Sugiyama

**Affiliations:** ^1^Department of Gastroenterology and Hematology, Faculty of Medicine, University of Toyama, 2630 Sugitani, Toyama 930-0194, Japan; ^2^Department of Gastroenterology, Kouseiren Takaoka Hospital, 5-10 Eiraku-cho, Takaoka, Toyama 933-8555, Japan; ^3^Department of Surgery, Takaoka City Hospital, 4-1 Takaramachi, Takaoka, Toyama 933-8550, Japan; ^4^Department of Surgical Pathology, University of Toyama, 2630 Sugitani, Toyama 930-0194, Japan

## Abstract

*Background*. S-1 plus cisplatin has been established to be standard first-line chemotherapy for advanced gastric cancer in Japan. The optimal second-line treatment refractory to S-1 plus cisplatin remains unclear. *Methods*. We retrospectively studied the efficacy, toxicity, and survival of irinotecan plus mitomycin C in patients with advanced gastric cancer refractory to a fluoropyrimidine plus cisplatin. *Results*. Twenty-four patients were studied. Prior chemotherapy was S-1 plus cisplatin in 15 patients, S-1 plus cisplatin and docetaxel in 8, and 5-fluorouracil plus cisplatin with radiotherapy in 1. The overall response rate was 17.4%. The median overall survival was 8.6 months, and the median progression-free survival was 3.6 months. Grade 3 or 4 toxicities included leukopenia (33%), neutropenia (50%), anemia (33%), thrombocytopenia (4%), anorexia (13%), diarrhea (4%), and febrile neutropenia (13%). *Conclusion*. A combination of irinotecan and mitomycin C is potentially effective in patients with advanced gastric cancer refractory to a fluoropyrimidine plus cisplatin.

## 1. Introduction

Gastric cancer remains one of the most important malignancies in Japan, with a mortality rate of about 50,000 deaths per 100,000 inhabitants, making it the second leading cause of cancer-related death [1]. Outcomes remain very poor for patients with unresectable or recurrent gastric cancer, although survival has been improved by systemic chemotherapy as compared with best supportive care alone [2–4]. Chemotherapy for advanced gastric cancer has not been standardized for a long time in Japan. The Japan Clinical Oncology Group (JCOG) 9205 trial was a three-arm phase III study that compared UFT (a combination of uracil and tegafur) plus mitomycin C with 5-fluorouracil (5-FU) plus cisplatin and with 5-FU alone as first-line chemotherapy. The response rates were 8.6%, 34.3%, and 11.4%, and the median survival times were 6.0 months, 7.3 months, and 7.1 months in the UFT plus mitomycin C group, 5-FU plus cisplatin group, and 5-FU group, respectively [5]. Consequently, 5-FU monotherapy has been used as a reference arm in subsequent clinical trials, and a fluoropyrimidine combined with cisplatin has become a recommended treatment. Recently, two important phase III studies of first-line chemotherapy for advanced gastric cancer have been reported. Subsequently, the JCOG9912 trial (5-FU versus irinotecan plus cisplatin versus S-1) showed that S-1 monotherapy was as effective as 5-FU monotherapy in terms of overall survival (11.4 to 10.8 months; *P* = 0.005 for noninferiority), whereas irinotecan plus cisplatin was not significantly superiority to 5-FU monotherapy with regard to overall survival (12.3 months; *P* = 0.0552) [6]. The SPIRITS study (S-1 versus S-1 plus cisplatin), conducted at nearly the same time, demonstrated that overall survival with S-1 plus cisplatin was superior to that with S-1 monotherapy (13.0 versus 11.0 months; *P* = 0.04) [7]. On the basis of these results, S-1 plus cisplatin was accepted to be standard therapy for advanced gastric cancer in Japan. Outside Japan, a randomized phase III trial compared irinotecan as second-line chemotherapy with best supportive care alone in patients with advanced gastric cancer. Irinotecan monotherapy was demonstrated to be superior to best supportive care in terms of overall survival, indicating that second-line chemotherapy is beneficial in this indication [8].

Irinotecan is a derivative of camptothecin that exerts antitumor activity by inhibiting DNA topoisomerase-1. Futatsuki et al. performed a phase II trial of second-line chemotherapy in Japanese patients with advanced gastric cancer. Irinotecan monotherapy had an overall response rate of 23% and a response rate of 20% in patients who had received prior treatment [9]. Irinotecan combined with drugs such as cisplatin or mitomycin C was then studied in several clinical trials. Boku et al. obtained an overall response rate of 48% and a response rate of 27% with irinotecan plus cisplatin in patients with metastatic gastric cancer who had received prior treatment [10]. However, there were high incidences of grade 4 neutropenia (57%) and grade 3-4 diarrhea (20%), suggesting that this regimen was too toxic. Yamao et al. performed a phase I/II study of irinotecan plus mitomycin C therapy and obtained an overall response rate of 50% and a response rate of 36% in patients with advanced gastric cancer who had received prior treatment [11]. This regimen was considered active as a second-line therapy. In the JCOG0109-DI trial, a phase II study in patients with fluoropyrimidine-resistant advanced gastric cancer, Hamaguchi et al. obtained an overall response rate of 29% and a median survival time of 10 months with irinotecan plus mitomycin C as second-line chemotherapy [12].

 Various phase II/III studies of second-line therapies for advanced gastric cancer have thus been conducted in Japan. However, many of these trials have involved fluoropyrimidine-refractory patients, and few studies have evaluated the effectiveness of second-line chemotherapy in patients who are refractory to a fluoropyrimidine combined with cisplatin. In the present study, we retrospectively investigated efficacy, toxicity, and survival in patients who received irinotecan plus mitomycin C after they were found to be refractory to combined chemotherapy with a fluoropyrimidine plus cisplatin, especially S-1 plus cisplatin, which is now the standard first-line treatment for advanced gastric cancer in Japan.

## 2. Patients and Methods

From November 2006 through March 2008, we studied 24 patients (22 men and 2 women) with advanced gastric cancer who received irinotecan plus mitomycin C as second-line treatment after failure to respond to first-line chemotherapy with a fluoropyrimidine plus cisplatin at Toyama University Hospital, Takaoka City Hospital, or Kouseiren Takaoka Hospital. Irinotecan (150 mg/m^2^) was given as a 90 min intravenous infusion, and mitomycin C (5 mg/m^2^) was given as an intravenous bolus on day 1 of a 14-day cycle. Treatment was repeated every 2 weeks until progressive disease, unacceptable toxicity, or patient refusal. The total administered dose of mitomycin C had to be less than 50 mg/m^2^ to prevent delayed cumulative toxicity, such as hemolytic uremic syndrome and pulmonary fibrosis. All patients received premedication with serotonin (5-hydroxytryptamine_3_) antagonists and dexamethasone.

 Tumor responses were evaluated according to the Response Evaluation Criteria in Solid Tumors (RECIST), version 1.0. Adverse events were graded according to the Common Terminology Criteria for Adverse Events (CTCAE), version 3.0. We measured survival from the date of initiating combined chemotherapy with irinotecan and mitomycin C. Actuarial curves for progression-free survival and overall survival were calculated with the Kaplan-Meier method.

## 3. Results

### 3.1. Patient Population

The patients' clinical characteristics are shown in Table 1. The median age was 63 years (range, 49–73). The Eastern Cooperative Oncology Group (ECOG) performance status was 0 or 1 in 21 patients and 2 in 3 patients. Lymph nodes, liver, and peritoneum were the most common sites of metastases. First-line chemotherapy was S-1 plus cisplatin in 15 patients and S-1 plus cisplatin and docetaxel in 8. The remaining patient, whose primary tumor was located at the esophagogastric junction, received 5-FU plus cisplatin and concomitant radiotherapy.

### 3.2. Treatment

In total, 144 courses of chemotherapy were administered. The median number of courses per patient was 5.5 (range, 2–10). Dose reduction was performed in 16 patients because of hematologic toxicity (12 patients), diarrhea (1 patient), or other reasons (3 patients).

### 3.3. Objective Response and Survival

**Figure 1:**
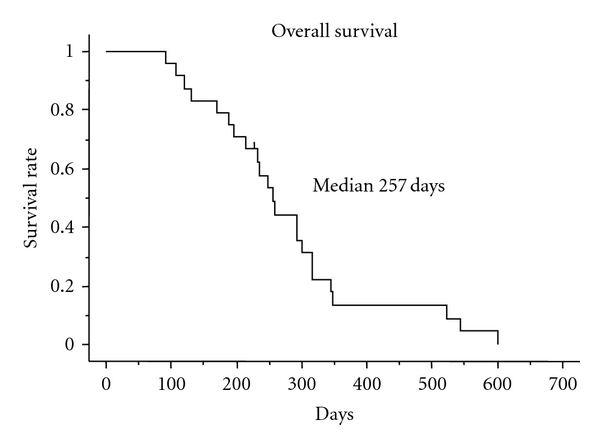
Overall survival of 24 patients.

**Figure 2:**
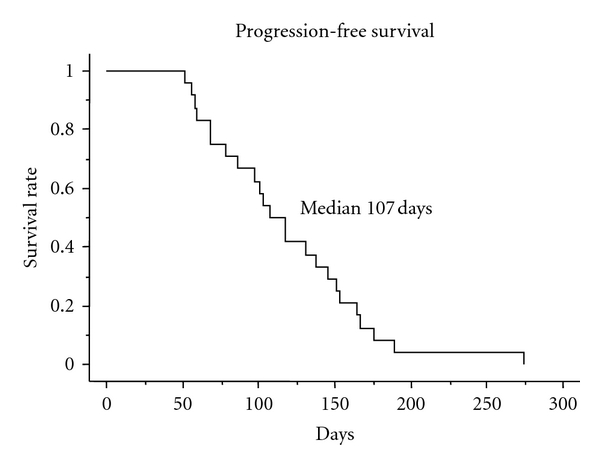
Progression-free survival of 24 patients.

Among the 23 patients in whom response was assessable, 4 had a partial response. The overall response rate was 17.4% (95% confidence interval (CI), 1.9% to 32.9%) (Table 2). The median overall survival was 8.6 months (Figure 1), and the median progression-free survival was 3.6 months (Figure 2).

### 3.4. Toxicity

Toxicity was assessable in all patients and is summarized according to grade in Table 3. All nonhematologic toxic effects were relatively mild (grade 1 or 2), except for grade 3 anorexia (13%), grade 3 diarrhea (4%), and grade 3 febrile neutropenia (13%). Hematologic toxicity included leukopenia (33%), neutropenia (50%), anemia (33%), and thrombocytopenia (4%). Seven patients had grade 4 neutropenia. Acute interstitial pneumonia occurred in one patient and responded to steroid treatment. There were no treatment-related deaths.

## 4. Discussion

 The JCOG9912 and SPIRITS trials established S-1 plus cisplatin as standard first-line chemotherapy for advanced gastric cancer in Japan [6, 7]. On the other hand, the FLAGS trial (S-1 plus cisplatin versus 5-FU plus cisplatin), a non-Asian global phase III study, showed that S-1 plus cisplatin was not superior to 5-FU plus cisplatin with regard to overall survival (8.6 versus 7.9 months; *P* = 0.20) [13]. Therefore, 5-FU plus cisplatin is still more frequently used than S-1 plus cisplatin for first-line treatment in the west. Moreover, another study reported that second-line treatment with irinotecan significantly prolonged overall survival as compared with best supportive care alone in patients with advanced gastric cancer: median survival was 123 days in the irinotecan group as compared with 72.5 days in the best supportive care group (*P* = 0.0027) [8]. Various studies have thus examined which regimens are effective as second-line chemotherapy after a fluoropyrimidine plus cisplatin, with the ultimate goal of improving outcomes in advanced gastric cancer.

 Phase II studies of irinotecan plus mitomycin C as a second-line chemotherapy for advanced gastric cancer have been reported. Bamias et al. evaluated the tolerance and efficacy of irinotecan (125 mg/m^2^) plus mitomycin C (5 mg/m^2^) given every 2 weeks to patients with advanced gastric cancer or colorectal cancer who had previously received 5-FU. The overall response rate was 12.5%, with a median progression-free survival of 5 months and a median overall survival of 8 months [14]. In a study performed by Giuliani et al., patients with advanced gastric cancer received irinotecan (150 mg/m^2^) on days 1 and 15 and mitomycin C (8 mg/m^2^) on day 1 of a 4-week cycle. The overall response rate was 32%, and the time to progression and overall survival were 4 months and 8 months, respectively [15]. Then, Hamaguchi et al. assessed the efficacy of irinotecan plus mitomycin C in patients with advanced gastric cancer, similar to our study. They obtained an overall response rate of 29%, a time to progression of 4.1 months, and an overall survival of 10 months, respectively. The response rate in our study (17.4%) was much lower, possibly because of differences in first-line chemotherapy received by the patients. In the study by Hamaguchi et al., most patients were given 5-FU-based monotherapy (i.e., 5-FU monotherapy, 40%; S-1 monotherapy, 33%; S-1 plus cisplatin, 13%; methotrexate plus 5-FU, 4%) [12]. In our study, all patients had previously received a fluoropyrimidine plus cisplatin. This and other differences in the background characteristics of the patients at the initiation of second-line chemotherapy might have thus reduced the response rate in our study. Moreover, median survival in our study was somewhat short, but we consider our survival data to be acceptable for second-line chemotherapy, given that the performance status of our patients (3 patients had a performance status of 2) was poorer than that of patients in previous studies.

Several studies of irinotecan-based second-line chemotherapy have been reported, although the backgrounds of the subjects differed considerably. The rate of response to irinotecan monotherapy was 20.0% [9], and the median survival was 123 days in one trial comparing irinotecan plus best supportive care with best supportive care alone [8]. Kim et al. obtained a response rate of 18.2% and a median time to progression and overall survival of 2.3 and 5.1 months, respectively, in a phase II study of a combination of irinotecan, continuous 5-FU, and leucovorin (FOLFIRI) in patients with advanced gastric cancer who had previously received a fluoropyrimidine-based regimen [16]. We consider our results to be at least comparable to the findings of these studies, and irinotecan plus mitomycin C does not require an intravenous port system.

Toxicity in our study was similar to that reported previously. The incidences of grade 3 or higher toxic effects were as follows: leukopenia, 33%; neutropenia, 50%; anemia, 33%; anorexia, 13%. The incidences of these adverse effects were consistent with those in a previous phase II study of biweekly mitomycin C and irinotecan (leukopenia, 29%; neutropenia, 53%; anemia, 13%; anorexia, 24%) [12]. Treatment was generally well tolerated, and adverse events were manageable.

 Third-line chemotherapy was given to 19 patients in our study, while 5 patients received best supportive care. Seventeen patients received paclitaxel (80 mg/m^2^ as a 90 min intravenous infusion) on days 1, 8, and 15 of a 28-day cycle. This regimen is frequently used for second-line [17, 18] as well as third-line chemotherapy [19]. In Japan, key drugs for the chemotherapeutic management of advanced gastric cancer are fluoropyrimidines (5-FU or S-1, etc.), cisplatin, irinotecan, and taxanes (docetaxel or paclitaxel). Currently, S-1 plus cisplatin is a standard first-line therapy, and irinotecan- or taxane-based regimens are often used for second-or third-line treatment [20–22]. We suppose that irinotecan-based regimens are better suited for second-line than third-line chemotherapy. Because irinotecan can cause severe toxic effects (ascites with carcinomatous peritonitis during progressive disease), it should be used at an earlier stage [19].

In conclusion, our results showed that combined chemotherapy with irinotecan and mitomycin C is effective in patients with disease that is refractory to a fluoropyrimidine plus cisplatin, especially S-1 plus cisplatin, which is currently the standard first-line regimen for advanced gastric cancer in Japan. Clinically, a combination of irinotecan and mitomycin C might be an effective treatment, contribute to the palliation or prevention of symptoms, prolong survival.

## Figures and Tables

**Table 1:** Patient characteristics.

	Number of patients
Sex	
Male	22
Female	2
Age (median)	63 (49–73)
Performance status (ECOG)	
0	9
1	12
2	3
Histology	
Intestinal	16
Diffuse	8
Site of metastasis	
Lymph nodes	22
Liver	14
Peritoneum	10
Lung	6
Prior chemotherapy	
S-1 + cisplatin	15
S-1 + cisplatin + docetaxel	8
5-FU + cisplatin + radiation therapy	1

ECOG, Eastern Cooperative Oncology Group; S-1, tegafur, gimeracil, oteracil potassium; 5-FU, 5-fluorouracil.

**Table 2:** Response (23 patients).

	CR	PR	SD	PD	RR
Overall	0	4	4	15	17.4% (95% CI, 1.9–32.9%)

CR: complete response; PR: partial response; SD: stable disease;

PD: progressive disease; RR: response rate; CI: confidence interval.

**Table 3:** Toxicity (24 patients).

Toxicity	Grade (CTCAE ver.3.0)				Grade 3/4 (%)
	1	2	3	4	
Hematologic					
Leukopenia	5	10	7	1	33
Neutropenia	3	8	5	7	50
Anemia	5	11	6	2	33
Thrombocytopenia	9	4	1	0	4
Nonhematologic					
Anorexia	10	7	3	0	13
Nausea/vomiting	5	1	0	0	0
Diarrhea	11	3	1	0	4
Fatigue	13	8	0	0	0
Febrile neutropenia	—	—	3	0	13
Pneumonitis	1	0	0	0	0

CTCAE: Common Terminology Criteria for Adverse Events.
